# Diagonal Earlobe Crease and the Risk of New-Onset Atrial Fibrillation After Cavotricuspid Isthmus Ablation in Patients with Typical Atrial Flutter

**DOI:** 10.3390/life16030508

**Published:** 2026-03-19

**Authors:** Moo-Nyun Jin, Young Ju Kim, Changho Song

**Affiliations:** 1 Department of Cardiology, Incheon Sejong Hospital, Incheon 21080, Republic of Korea; 2 Department of Internal Medicine, Ewha Womans University College of Medicine, Seoul 07985, Republic of Korea; 3 Jinsim Heart Rhythm Laboratory, Seoul 05553, Republic of Korea; 4 Department of Cardiology, Catholic Kwandong University International St. Mary’s Hospital, Incheon 22711, Republic of Korea; 5 Division of Cardiology, BHS Hanseo Hospital, Busan 48253, Republic of Korea

**Keywords:** atrial fibrillation, atrial flutter, catheter ablation, earlobe crease, Frank’s sign, risk prediction, cardiovascular risk factors

## Abstract

**Background:** Atrial fibrillation (AF) frequently develops in patients with atrial flutter (AFL), even after successful cavotricuspid isthmus (CTI) ablation. Identifying simple clinical markers for early detection is crucial. Diagonal earlobe crease (ELC), also known as Frank’s sign, has been proposed as a marker of aging and cardiovascular risk. This study investigates the association between ELC and the risk of new-onset AF following CTI ablation in patients with AFL. **Methods:** We conducted a retrospective cohort study of 292 patients without a prior history of AF who underwent CTI ablation for typical AFL between 2015 and 2024. The presence of ELC was assessed at baseline CTI ablation. The primary outcome was the occurrence of new-onset AF during follow-up, stratified according to the presence of ELC. The median follow-up duration was 49 months, with a minimum follow-up of 6 months. **Results:** Among the 292 patients, 72 (24.7%) exhibited ELC. Patients with ELC were older (59 ± 11 years vs. 55 ± 14 years, *p* = 0.05). During the follow-up period, new-onset AF occurred in 31 patients with ELC (43.1%) and 65 patients without ELC (29.5%) (*p* = 0.03). Kaplan–Meier analysis demonstrated that the occurrence of AF was significantly higher in the ELC group than in the non-ELC group (log-rank test, *p* = 0.013). Multivariate analysis revealed that ELC was independently associated with an increased risk of AF (hazard ratio 1.67, 95% confidence interval 1.03–2.72, *p* = 0.039). **Conclusions:** The presence of ELC is associated with a higher risk of new-onset AF following CTI ablation in patients with AFL. ELC may serve as a simple, non-invasive clinical marker to identify patients who may benefit from closer rhythm surveillance after AFL ablation.

## 1. Introduction

Atrial flutter (AFL) is a common supraventricular tachyarrhythmia characterized by rapid and regular atrial contractions. Cavotricuspid isthmus (CTI) ablation is an effective treatment for typical AFL, achieving high success rates in restoring sinus rhythm [1,2]. However, a significant proportion of patients develop atrial fibrillation (AF) following successful CTI ablation, with reported incidence rates ranging from 30% to 60% within a few years of follow-up [3]. Early detection and management of AF are crucial, as undiagnosed AF may lead to adverse outcomes, including stroke, heart failure and dementia [4]. Identifying simple and non-invasive clinical markers that may help refine post-ablation surveillance strategies is therefore of clinical interest [5].

The diagonal earlobe crease (ELC), also known as Frank’s sign, is a physical marker characterized by a diagonal fold or wrinkle extending obliquely from the tragus towards the earlobe’s edge. First described by Sanders T. Frank in 1973 [6], ELC has been associated with various cardiovascular diseases, including coronary artery disease, peripheral vascular disease, and cerebrovascular events. The etiopathogenic link between ELC and atherosclerotic disease remains incompletely understood. It has been proposed that ELC may result from microvascular damage leading to degeneration of elastic fibers in the earlobe, which could reflect similar pathological changes in the coronary arteries [7]. ELC also occurs more commonly with advancing age and is reported to be a simple visible cutaneous marker of accelerated biological aging. This is supported by its rarity in pediatric populations [8], its association with telomere shortening [9], and elevated levels of inflammatory and oxidative stress biomarkers in individuals exhibiting ELC [10].

Underlying mechanisms linking ELC to cardiovascular pathology remain speculative but may involve systemic atherosclerosis, inflammatory stress, and aging-related degenerative changes that are also relevant to atrial remodeling. AF is the most common age-related heart rhythm disorder. AF and ELC share common risk factors, with advanced age being associated with a higher risk of AF and ELC development [10,11,12]. Given the role of aging and systemic degenerative changes in the pathophysiology of AF, we hypothesized that the presence of ELC might be associated with an increased risk of AF development after AFL ablation. However, research exploring the relationship between ELC and AF, particularly in patients undergoing CTI ablation for typical AFL, remains limited. This study aims to evaluate the potential predictive value of ELC for new-onset AF in patients undergoing CTI ablation for AFL.

## 2. Materials and Methods

### 2.1. Study Population

**Figure 1:**
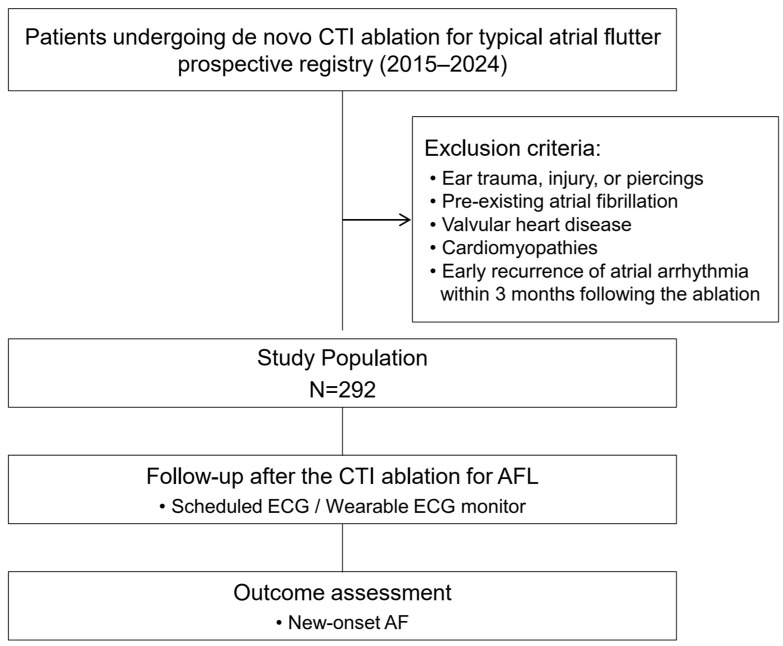
Flow diagram of the study population and design.

The study population consisted of consecutive patients aged <65 years who underwent de novo successful catheter ablation for typical AFL at hospitals in the Republic of Korea between 2015 and 2024. The age restriction was applied to minimize the confounding effects of advanced age on the assessment of ELC as a marker of biological aging. Typical AFL was diagnosed based on surface electrocardiographic findings, including readily visible negative flutter waves in the inferior leads and positive flutter waves in lead V1 with a regular atrial rate [13]. The exclusion criteria were as follows: (1) a history of ear trauma, injury, or piercings; (2) prior documented AF; (3) previous open-heart surgery; (4) moderate-to-severe valvular heart disease; (5) cardiomyopathies, including hypertrophic cardiomyopathy, cardiac amyloidosis, and arrhythmogenic ventricular cardiomyopathy, as well as hereditary channelopathies; and (6) early recurrence of atrial arrhythmia (atrial flutter or atrial fibrillation) within 3 months following AFL ablation. Participants were consecutively enrolled in a prospective institutional registry of patients undergoing de novo CTI ablation for typical AFL. At the time of enrollment, the presence of ELC was assessed by trained healthcare providers through direct clinical inspection, with standardized ear photographs available for a subset of patients. The present study represents a retrospective analysis of these prospectively collected registry data (Figure 1). Written informed consent was obtained from all patients. The study protocol was approved by the Institutional Review Board of Ewha Womans University Medical Center (IRB No. 2024-01-008) on 24 January 2024.

### 2.2. Electrophysiology Study and Catheter Ablation

Antiarrhythmic drugs were discontinued for at least five half-lives before the procedure. Electrophysiology studies were performed in the postabsorptive state. Diagnostic catheters were positioned as follows: (1) a duodecapolar catheter was positioned in the right atrium, parallel to the tricuspid annulus so that the distal pole was located in medial region of the CTI, (2) a decapolar catheter was inserted within the coronary sinus, with the proximal bipole located at the ostium, and (3) quadripolar catheter was positioned at the His bundle and right ventricle. Catheter ablation was performed using standard radiofrequency energy. Linear ablation was initiated along the ventricular aspect of the CTI, with sequential lesions delivered from the tricuspid annulus to the inferior vena cava. Procedural success was defined as the achievement of bidirectional conduction block across the CTI, confirmed using differential pacing maneuvers.

### 2.3. Assessment of ELC

**Figure 2:**
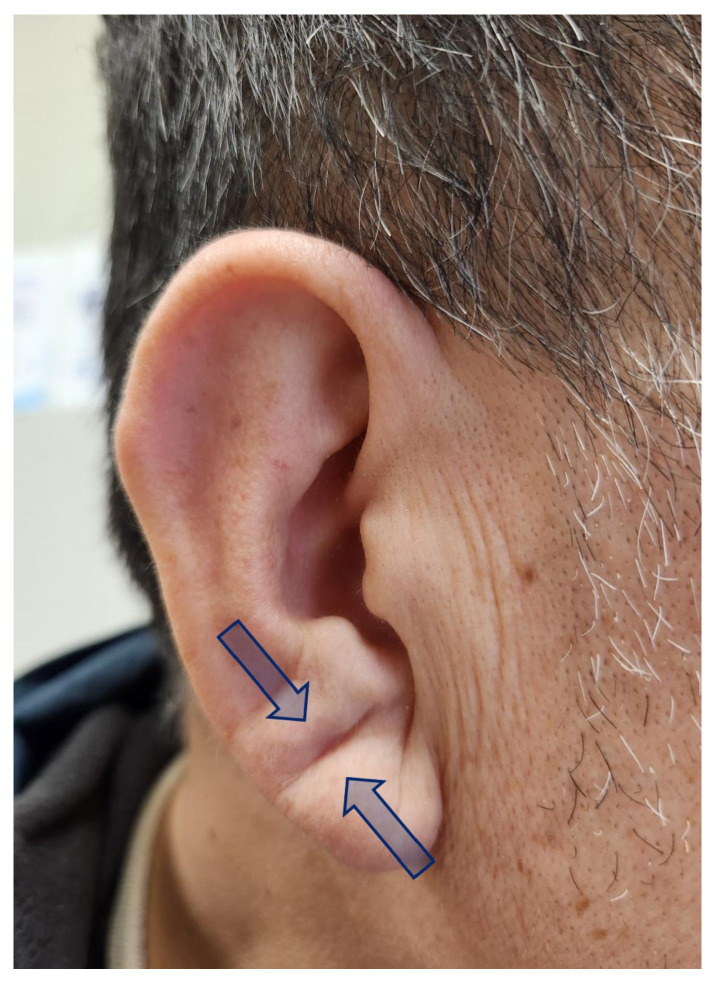
A representative example of an earlobe crease, indicated by arrows.

At the time of baseline AFL ablation, the presence of diagonal ELC was assessed through direct clinical inspection, and standardized photographs of both ears were obtained with participants in a seated position. The images were reviewed by a trained healthcare provider who was blinded to clinical outcomes to assess the presence of ELC. ELC was defined according to previously published criteria [14]. Specifically, a positive ELC was defined as a clearly visible deep furrow or wrinkle extending diagonally from the tragus toward the outer border of the earlobe without interruption (Figure 2). The presence of such a crease in either one or both ears was classified as ELC-positive.

### 2.4. Study Outcome and Follow-Up

The primary study outcome was the occurrence of new-onset AF during follow-up. AF was defined as an episode of absolutely irregular RR intervals lasting >30 s, without discernible P waves [15]. AF surveillance was performed using scheduled 12-lead electrocardiography at 1, 3, 6, and 12 months after enrollment. In addition, 24 h Holter monitoring or single-channel wearable electrocardiogram (ECG) monitoring was conducted at 6 and 12 months. After the first year of follow-up, 12-lead ECGs were performed at 6-month intervals or when patients reported symptoms suggestive of arrhythmia. The planned study duration was 60 months, with a minimum follow-up period of 6 months.

### 2.5. Statistical Analysis

Continuous variables are presented as means with standard deviations or as medians with interquartile ranges, as appropriate according to data distribution. Categorical variables are expressed as counts and percentages. To minimize selection bias and account for potential confounding inherent to the observational study design, baseline characteristics were balanced using propensity score matching. Propensity scores were estimated using a non-parsimonious multivariable logistic regression model to calculate the probability of ELC presence. Covariates included age, sex, hypertension, diabetes mellitus, heart failure, prior stroke or transient ischemic attack, and dyslipidemia, selected based on their established relevance as cardiovascular risk factors [16,17]. Matching was performed in a 1:1 ratio using optimal matching without replacement, with a caliper width of 0.2 standard deviations of the logit of the propensity score to ensure adequate balance between groups. Baseline characteristics and comorbidities were compared after matching. Continuous variables were compared using Student’s *t*-test or the Mann–Whitney U test, as appropriate, while categorical variables were analyzed using the chi-square test or Fisher’s exact test. Time-to-event analyses were performed to evaluate study outcomes. Hazard ratios (HRs) and 95% confidence intervals (CIs) were estimated using Cox proportional hazards regression models applied to the matched cohort. Multivariable Cox proportional hazards models were constructed using clinically relevant covariates selected a priori based on established associations with atrial fibrillation risk [15,18,19]. Kaplan–Meier survival curves were constructed to illustrate differences in event-free survival according to ELC status, and comparisons between groups were performed using the log-rank test. All statistical tests were two-sided, and a *p* value < 0.05 was considered statistically significant. All analyses were conducted using R software (version 4.3; R Foundation for Statistical Computing, Vienna, Austria).

## 3. Results

### 3.1. Baseline Characteristics

A total of 292 participants younger than 65 years of age (mean age: 57.8 ± 10.5 years) without a prior history of atrial fibrillation at baseline were enrolled in the study. Among the 292 patients, 72 (24.7%) exhibited ELC. Baseline characteristics of the overall cohort and of the propensity-score-matched populations are shown in Table 1. In the overall cohort, patients in the ELC group were older than those in the non-ELC group. Other baseline characteristics, including sex distribution, comorbidities, echocardiographic parameters, and follow-up duration, were comparable between groups. After propensity score matching, baseline demographic characteristics, cardiovascular comorbidities, and echocardiographic findings were well balanced between the ELC and non-ELC groups, with no statistically significant differences observed.

### 3.2. AF Incidence According to ELC Status

**Figure 3:**
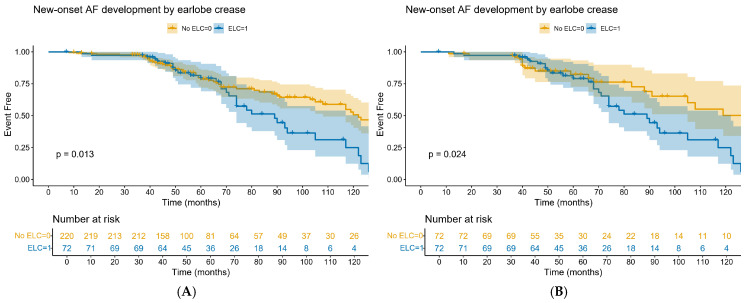
Kaplan–Meier curves showing atrial-fibrillation-free survival according to the presence of diagonal earlobe crease (ELC) in the overall population (**A**) and the propensity-score-matched population (**B**). Shaded areas represent 95% confidence intervals. The numbers at risk at selected time points are shown below the plots.

During a median follow-up of 49 months (interquartile range, 40–80 months), new-onset AF occurred more frequently in the ELC group than in the non-ELC group in the overall population (31 patients [43.1%] vs. 65 patients [29.5%], *p* = 0.03). In the propensity-score-matched cohort, the incidence of AF remained numerically higher in the ELC group compared with the non-ELC group (43.1% vs. 33.3%), although this difference did not reach statistical significance (*p* = 0.23). Kaplan–Meier analysis showed a higher cumulative incidence of AF in patients with ELC than in those without ELC in the overall population (Figure 3A; log-rank *p* = 0.013). This association remained consistent in the propensity-score-matched population (Figure 3B; log-rank *p* = 0.024).

In Cox proportional hazards regression analyses, the presence of ELC was associated with an increased risk of AF in both the overall and propensity-score-matched populations. In the multivariable model for the overall population, ELC was independently associated with AF development (hazard ratio [HR], 1.67; 95% confidence interval [CI], 1.03–2.72; *p* = 0.039). In the propensity-score-matched population, the association between ELC and AF remained significant after multivariable adjustment (HR, 2.13; 95% CI, 1.13–3.99; *p* = 0.019) (Table 2).

## 4. Discussion

The present study investigated the association between ELC and the development of new-onset AF following CTI ablation in patients with AFL. The main findings of this study were as follows: (1) patients with ELC who underwent CTI ablation for AFL had a significantly higher risk of developing new-onset AF than those without ELC; and (2) the presence of ELC independently predicted the development of new-onset AF after CTI ablation in patients with AFL. Taken together, these findings suggest that ELC may serve as a readily observable clinical marker associated with an increased risk of AF in patients with AFL following CTI ablation.

ELC, also known as “Frank’s sign,” is a wrinkle or fold extending diagonally from the tragus toward the border of the earlobe. It was first described as a clinical sign associated with coronary artery disease by Sanders T. Frank in 1973 [6]. Subsequent studies have consistently demonstrated an association between ELC and atherosclerotic cardiovascular disease. Accordingly, ELC has been considered a visible marker of underlying coronary atherosclerosis and systemic vascular pathology [7]. ELC has also been linked to biomarkers of inflammation and oxidative stress, both of which play central roles in age-related cardiovascular disorders [10]. AF, the most common age-related arrhythmia, shares many risk factors with ELC, including hypertension, diabetes, obesity, dyslipidemia, and metabolic syndrome [15]. However, the usefulness of ELC as a risk marker for AF remains incompletely defined, particularly in clinical settings in which AF develops after an index arrhythmia such as typical AFL. Despite accumulating epidemiological evidence linking ELC to adverse cardiovascular outcomes, the biological basis underlying its association with AF—especially following CTI ablation—has not yet been fully elucidated.

Several plausible pathophysiological mechanisms may explain the observed relationship between ELC and AF. ELC has been widely regarded as a surrogate marker of systemic vascular aging and subclinical atherosclerosis, reflecting microvascular dysfunction, chronic inflammation, and degeneration of elastic fibers in end-arterial territories [10,20]. Importantly, these pathological processes are not confined to the peripheral vasculature but may also involve the atrial myocardium, thereby contributing to the formation of an arrhythmogenic substrate [12].

AF is increasingly recognized as a manifestation of atrial cardiomyopathy rather than a purely electrical disorder and is characterized by structural remodeling, interstitial fibrosis, endothelial dysfunction, and microvascular ischemia [21]. Notably, many of the biological processes implicated in atrial remodeling—including oxidative stress, inflammatory activation, and impaired endothelial homeostasis—have also been associated with the presence of ELC [10,12]. Previous studies have shown that individuals with ELC exhibit elevated circulating inflammatory and oxidative stress biomarkers, such as pentraxin-3 and malondialdehyde-modified low-density lipoprotein, even after adjustment for conventional cardiovascular risk factors [10]. In addition, reduced levels of endothelial-protective peptides, including adropin and irisin, have been reported in patients with ELC, suggesting impaired nitric oxide signaling and endothelial dysfunction as potential shared mechanisms [22].

These pathophysiological pathways may be relevant to the development of AF. Chronic low-grade inflammation and oxidative stress promote atrial fibrosis, electrical heterogeneity, and conduction slowing, thereby facilitating both the initiation and maintenance of AF [11,21]. From this perspective, ELC may represent an external phenotypic marker of a systemic and atrial pro-arrhythmogenic milieu rather than a condition-specific sign. These interpretations should be considered hypothesis-generating rather than definitive mechanistic explanations, as the present study was not designed to directly investigate the biological mechanisms linking ELC and AF. The persistence of the association between ELC and AF in multivariable analyses, even after adjustment for age and traditional cardiovascular risk factors, supports the concept that ELC may capture aspects of biological aging or atrial vulnerability that are not fully reflected by conventional clinical or echocardiographic parameters.

In this context, the relatively stronger association observed for ELC compared with some conventional clinical or echocardiographic variables may reflect its role as a phenotypic marker of cumulative cardiovascular aging. While parameters such as atrial size represent a single structural measurement at a given time point, ELC may capture broader aspects of systemic vascular dysfunction, inflammatory activation, and oxidative stress that contribute to atrial remodeling. Therefore, the presence of ELC may indicate individuals with a more advanced pro-arrhythmogenic substrate that may not be fully reflected by conventional risk factors alone.

In patients with typical AFL, CTI ablation effectively abolishes the macro-reentrant circuit responsible for the arrhythmia but does not modify the underlying atrial substrate [3]. Growing evidence suggests that AF detected after successful CTI ablation frequently represents the unmasking of pre-existing atrial disease rather than a direct consequence of the ablation procedure itself [5,23]. Editorials and long-term follow-up studies have emphasized that the incidence of AF continues to increase over time—particularly with more intensive rhythm monitoring—highlighting the progressive nature of atrial disease in this population [24]. Within this context, the presence of ELC may identify patients with more advanced atrial remodeling at baseline, thereby predisposing them to AF after AFL ablation.

From a clinical perspective, early identification of patients at increased risk for AF following AFL ablation is of considerable importance. AF is associated with a substantially higher risk of thromboembolic events, heart failure, cognitive impairment, and mortality compared with AFL and often necessitates a distinct therapeutic approach, including long-term anticoagulation. Nevertheless, current guidelines do not provide specific recommendations for AF surveillance after CTI ablation, and routine long-term continuous rhythm monitoring is not feasible for all patients [15,18]. In this setting, a simple, non-invasive, and cost-free physical finding such as ELC may offer incremental value as a readily observable marker for risk enrichment. Rather than serving as a diagnostic criterion, ELC may function as a clinical phenotype that prompts heightened vigilance and consideration of individualized rhythm surveillance strategies.

Several limitations of the present study should be acknowledged. First, the observational and retrospective nature of the study precludes definitive causal inference, and residual confounding cannot be entirely excluded despite comprehensive multivariable adjustment and propensity score matching. Furthermore, the study population was restricted to patients younger than 65 years in order to minimize the confounding influence of advanced age when evaluating ELC as a marker of biological aging. However, this age restriction may limit the generalizability of the findings to older populations in whom AF is more prevalent. Nevertheless, multivariable adjustment and propensity score matching were applied to minimize potential selection bias and to ensure balanced comparisons between groups. Second, although AF surveillance was conducted using scheduled electrocardiography, Holter monitoring, wearable ECG devices, and symptom-driven evaluations, continuous long-term rhythm monitoring was not systematically implemented. Consequently, asymptomatic or short-duration AF episodes may have been under detected. Third, ELC was assessed at baseline using standardized evaluation, and longitudinal changes in ELC status were not examined. This approach reflects the primary aim of the study, which was to evaluate the prognostic significance of ELC as a readily observable baseline clinical marker rather than to investigate its temporal evolution. In addition, ELC assessment was performed by a single evaluator, and interobserver reliability was not formally evaluated, which may introduce potential measurement variability. Because this study was conducted in a Korean population, the generalizability of these findings to other ethnic or geographic populations should be interpreted with caution. Finally, although a broad range of clinical and echocardiographic variables were included in the analyses, unmeasured factors—such as genetic predisposition, atrial fibrosis burden assessed by advanced imaging, or circulating biomarkers—could not be fully accounted for. Accordingly, the mechanistic links between ELC and AF remain speculative. Future prospective multicenter studies incorporating continuous rhythm monitoring and mechanistic assessments will be required to further elucidate the pathophysiological relationship between ELC and atrial arrhythmogenesis.

## 5. Conclusions

The presence of a diagonal ELC is independently associated with an increased risk of new-onset AF after CTI ablation in patients with typical AFL. As a simple, non-invasive, and readily observable physical finding, ELC may offer incremental clinical value for identifying patients who warrant closer rhythm surveillance following AFL ablation. Further prospective studies are needed to clarify its role in post-ablation risk stratification and to determine whether earlier detection of AF in this population can translate into improved clinical outcomes.

## Figures and Tables

**Table 1:** Baseline characteristics of the overall and propensity-score-matched populations.

Baseline Characteristics	Total Population			PS-Matched (1:1) Population		
	ELC Group (*n* = 72)	Non-ELC Group (*n* = 220)	*p* Value	ELC Group (*n* = 72)	Non-ELC Group (*n* = 72)	*p* Value
Age	59.0 ± 11.1	55.1 ± 14.1	0.05	59.0 ± 11.1	59.2 ± 9.4	0.89
Sex (Female)	11 (15.3%)	47 (21.4%)	0.32	11 (15.3%)	11 (15.3%)	1.00
BMI (kg/m^2^)	23.8 ± 2.9	24.8 ± 3.2	0.63	23.8 ± 2.9	24.0 ± 2.9	0.59
***C*** ***omorbidities***						
Heart failure	1 (1.4%)	14 (6.4%)	0.17	1 (1.4%)	2 (2.8%)	1.00
Hypertension	33 (45.8%)	72 (32.7%)	0.07	33 (45.8%)	30 (41.7%)	0.74
Diabetes	17 (23.6%)	39 (17.7%)	0.26	17 (23.6%)	17 (23.6%)	1.00
Dyslipidemia	10 (13.9%)	22 (10.0%)	0.33	10 (13.9%)	9 (12.5%)	1.00
History of stroke/TIA	4 (5.6%)	11 (5.0%)	1.00	4 (5.6%)	4 (5.6%)	1.00
Vascular disease	9 (12.5%)	27 (12.3%)	0.91	9 (12.5%)	11 (15.3%)	0.81
CHA_2_DS_2_VAS_C_ score	1 (1–2)	1 (0–2)	0.39	1 (1–2)	1 (0–2)	0.76
***Echocardiogram***						
Left ventricular EF (%)	60.3 ± 10.1	61.6 ± 10.4	0.62	60.3 ± 10.1	61.5 ± 10.3	0.74
LAVI (mL/m^2^)	32.6 ± 10.7	35.4 ± 11.4	0.20	32.6 ± 10.7	35.8 ± 11.5	0.37
LA dimension (mm)	41.5 ± 5.1	42.0 ± 6.5	0.50	41.5 ± 5.1	42.2 ± 6.9	0.43

Values are expressed as n(%), means ± standard deviations, or medians (interquartile ranges), as appropriate. BMI, body mass index; EF, ejection fraction; ELC, diagonal earlobe crease; LA, left atrium; LAVI, left atrial volume index; PS, propensity score; TIA, transient ischemic attack.

**Table 2:** Cox proportional hazards model for the prediction of AF development.

	Total Population				PS-Matched (1:1) Population			
	Univariate Analysis		Multivariate Analysis *		Univariate Analysis		Multivariate Analysis *	
	HR	*p* Value	HR	*p* Value	HR	*p* Value	HR	*p* Value
Age	1.02 (1.00–1.03)	0.041	1.01 (0.99–1.02)	0.519	1.07 (1.02–1.12)	0.040	1.00 (0.97–1.02)	0.783
Female	0.44 (0.23–0.83)	0.011	0.61 (0.29–1.27)	0.185	0.63 (0.25–1.58)	0.324	0.60 (0.23–1.51)	0.276
Heart failure	0.72 (0.23–2.29)	0.581			1.81 (0.24–13.46)	0.563		
Hypertension	1.12 (0.72–1.74)	0.603			1.05 (0.59–1.84)	0.878		
Diabetes	1.13 (0.67–1.90)	0.654			1.74 (0.84–3.58)	0.137		
History of stroke/TIA	1.58 (0.73–3.43)	0.247			2.10 (0.64–6.83)	0.219		
Vascular disease	1.06 (0.57–1.94)	0.861			1.11 (0.55–2.28)	0.782		
Left ventricular EF (%)	1.00 (0.97–1.02)	0.711			0.99 (0.96–1.03)	0.695		
LAVI (mL/m^2^)	1.00 (0.99–1.02)	0.806			1.01 (0.98–1.04)	0.488		
LA dimension (mm)	1.03 (1.00–1.07)	0.069	1.03 (1.00–1.07)	0.064	1.03 (0.99–1.08)	0.138	1.04 (1.00–1.09)	0.058
ELC	1.77 (1.13–2.78)	0.013	1.67 (1.03–2.72)	0.039	1.96 (1.08–3.55)	0.026	2.13 (1.13–3.99)	0.019

* Multivariable Cox proportional hazards models were adjusted for clinically relevant covariates selected a priori. For clarity, only selected variables are presented in the table. EF, ejection fraction; ELC, diagonal earlobe crease; HR, hazard ratio; LA, left atrium; LAVI, left atrial volume index; PS, propensity score; TIA, transient ischemic attack.

## Data Availability

Data are available from the authors upon reasonable request with permission of the Institutional Review Board. The data are not publicly available due to privacy and ethical restrictions.

## Abbreviations

The following abbreviations are used in this manuscript:

AF	Atrial fibrillation
AFL	Atrial flutter
BMI	Body mass index
CI	Confidence interval
CTI	Cavotricuspid isthmus
ECG	Electrocardiogram
EF	Ejection fraction
ELC	Earlobe crease
HR	Hazard ratio
LA	Left atrium
LAVI	Left atrial volume index
PS	Propensity score
TIA	Transient ischemic attack
